# An Algorithmic Framework for Multiobjective Optimization

**DOI:** 10.1155/2013/859701

**Published:** 2013-12-29

**Authors:** T. Ganesan, I. Elamvazuthi, Ku Zilati Ku Shaari, P. Vasant

**Affiliations:** ^1^Department of Chemical Engineering, University Technology Petronas, 31750 Tronoh, Perak, Malaysia; ^2^Department of Electrical & Electronic Engineering, University Technology Petronas, 31750 Tronoh, Perak, Malaysia; ^3^Department of Fundamental & Applied Sciences, University Technology Petronas, 31750 Tronoh, Perak, Malaysia

## Abstract

Multiobjective (MO) optimization is an emerging field which is increasingly being encountered in many fields globally. Various metaheuristic techniques such as differential evolution (DE), genetic algorithm (GA), gravitational search algorithm (GSA), and particle swarm optimization (PSO) have been used in conjunction with scalarization techniques such as weighted sum approach and the normal-boundary intersection (NBI) method to solve MO problems. Nevertheless, many challenges still arise especially when dealing with problems with multiple objectives (especially in cases more than two). In addition, problems with extensive computational overhead emerge when dealing with hybrid algorithms. This paper discusses these issues by proposing an alternative framework that utilizes algorithmic concepts related to the problem structure for generating efficient and effective algorithms. This paper proposes a framework to generate new high-performance algorithms with minimal computational overhead for MO optimization.

## 1. Introduction

Many real-world problems can be reduced to scenarios that have a single aim or objective. However, it is difficult in some instances to incorporate all the aspects of the problem into a single objective function. Thus, a good way to go about incorporating multiple aspects is by defining multiple objectives and including them into the problem formulation. A mathematical representation of a multiobjective (MO) problem is as follows:

(1) Max⁡/Min⁡xu∈X⁡Fk(xu)subject  to X={xu:g(xu)=0; h(xu)≤0,u∈[1,l], k∈[1,p]},

where the indexes *u* and *k* denote the individual decision variables and the objective functions, *g*(*x*_*u*_) and *h*(*x*_*u*_) are the equality and inequality constraints, *l* is the maximum number of decision variables, and *p* is the maximum number of objective functions.

In recent times, many concerns have been raised when dealing with emerging technologies in industrial and engineering optimization which present themselves in a multiobjective (MO) setting [1–5]. Some of the issues are lack of computational power, insufficient computational techniques, and poor quality of obtained solution. Strategies in MO optimization can be rudimentarily classified into three groups. The first group is methods that use the concept of Pareto optimality to trace the nondominated solutions at the Pareto curve, for instance, in Strength Pareto Evolutionary Algorithm (SPEA) by Zitzler and Thiele [6] and Nondominated Sorting Genetic Algorithm II (NSGA-II) by Deb et al. [7]. The second class of techniques are known as the weighted (or scalarization) techniques. During the application of these methods, the objective functions are aggregated into a single weighted function which is then solved for various scalar (weight) values. Some well-known scalarization techniques include the weighted sum method [8] and normal-boundary intersection method (NBI) [9]. Using these techniques, the scalars (or weights) are used to consign relative tradeoffs to the objectives by the aggregation procedure. Hence, alternative near-optimal solution options are generated for various values of the scalars. The third group of techniques is the decomposition approaches. These approaches solve MO problems by simplifying the original problem into neighbouring subproblems. Among notable techniques which involve decomposition formulations are the MO evolutionary algorithm based on decomposition (MOEA/D) [10] and genetic algorithm with decomposition procedures [11].

In MO optimization problems, determining the most efficient solution set can be a very daunting process. Many varieties of concepts (such as diversity and convergence) have been proposed in the past [12]. These ideas were then used as indicators to evaluate solution sets produced by the optimization algorithm [12]. Such evaluations were then used to benchmark the algorithm's performance. These concepts unfortunately could not absolutely state and rank the superiority of solution sets produced by an algorithm against other such sets by other algorithms. Besides, the size of the Pareto frontiers is often directly proportional to the problem size. Since many industrial problems involve continuous functions (objectives), the Pareto frontiers obtained are infinite. Hence, such solutions are computationally impossible. The goal in such scenarios is usually to obtain a good approximation of the Pareto frontier.

The only concept that can be used generally for the overall ranking of solution sets is the idea of “Pareto dominance” [13]. The hypervolume indicator (HVI) [13] is a set measure reflecting the volume enclosed by a Pareto front approximation and a reference set (see [14–16]). The HVI guarantees strict monotonicity regarding Pareto dominance [17, 18]. This makes the ranking of solution sets and hence algorithms possible for any given MO problem. Nevertheless, other forms of metrics have also been developed and widely employed for benchmarking solution quality in MO optimization problems such as the convergence metric [19], diversity metric [20], and the HVI [21].

Over the past years, metaheuristic techniques have been applied with increasing frequency to industrial MO optimization problems. Some of the most effective metaheuristic techniques are the ones that spring from evolutionary and swarms approaches. One such evolutionary approach is the genetic algorithm (GA), introduced by Holland in the nineties [22]. GAs belongs to the group of stochastic search methods such as simulated annealing [23] and some forms of branch and bound. While most stochastic search techniques operate on a distinct solution for a particular problem, GAs operates on a population of solutions. In recent times, GAs have been widely applied in industrial scenarios (see [24–26]). Differential evolution (DE) is also a population-based evolutionary algorithm that has been derived from genetic algorithms (GA) [22]. DE was developed in the nineties by Storn and Price [27]. DE has been used extensively to solve problems which are nondifferentiable, noncontinuous, nonlinear, noisy, flat, multidimensional, and having many local minima, constraints, or high degree of stochasticity. Lately, DE has been applied to a variety of areas including optimization problems in chemical and process engineering [28–30].

One of the most popular swarm-based optimization approaches is the particle swarm optimization (PSO) algorithm. This optimization method was developed based on the movement and intelligence of swarms. PSO was developed by Kennedy and Eberhart [31] in 1995. Lately, PSO has been applied to a variety of areas including optimization problems in engineering [32] as well as economic dispatch problems. Another famed swarm approach is the gravitational search algorithm (GSA). This method introduced recently by Rashedi et al. [33] is currently among the most applied metaheuristic techniques in industrial optimization. GSA belongs to the group of swarm-based stochastic search methods (such as particle swarm optimization (PSO) [31] and cuckoo search algorithm (CSA) [34]). GSA operates on a population of solutions based on Newtonian law of gravity and mass interactions. This algorithm regards agents as objects consisting of different masses. In recent times, GSA has been broadly applied in many industrial settings (see [35]).

In a nutshell, currently MO optimization is done with the aid of metaheuristic algorithms, scalarization techniques, and nondominated Pareto optimal solution tracing. Besides, metric analysis is also employed to gauge the quality of the solutions obtained by any given algorithm. This paper aims to propose a philosophical framework that uses the information from metric analysis to generate high-performance and computationally efficient algorithms that produce high-quality solutions for MO optimization problems.

This paper is organized as follows: Section 2 provides a brief review on some previous works on industrial applications of MO optimization techniques, while Section 3 presents an overview on some current issues arising in MO optimization and the proposed methodology for MO optimization.  Section 4 includes some mathematical foundations with regard to the definitions and assumptions used in the proposed framework.  Section 5 provides some numerical experiments performed by the application of the proposed framework. Finally, this paper ends with some concluding remarks.

## 2. MO Techniques in Real-World Application

This section provides a review on various research works on the applications of MO optimization techniques ranging from general industrial applications and power systems to chemical processes. Over the past years, MO optimization has been introduced and applied into many real-world industrial scale problems. Some of these developments are presented in Table 1.

From previous research works presented in Table 1, it can be clearly seen that MO optimization has gradually spread towards many industrial applications. However, it can also be observed that in most cases only biobjective MO problem is considered. This may be due to fact that MO optimization is a new and upcoming field and only in recent times (with the aid of modern computational power) has this field spread its horizons into real-world industrial applications.

## 3. Proposed Framework

In the last ten to fifteen years, many industrial processes are seen from the MO optimization standpoint. In most cases, these processes are modelled with two or less objectives (see [36–38]). In such cases, other objectives are assumed to be a constant or treated as constraints. This is due to the lack of computational techniques and means of analysis for solving problems involving more than two objectives.

Subsequently many methods for Pareto frontier construction have been proposed in the past (see [4, 9]). However, very few studies have been conducted on the effects of convergence/diversity of the solution set (with respect to the problem's objective space) on the degree of Pareto dominance produced by any given MO algorithm.

Another issue is the philosophy of hybridization. Algorithm hybridization is a good approach to enhance the performance of any given algorithm (see [39, 40]). However, due to the increase in algorithmic complexity, this approach causes the algorithm to be computationally expensive and inefficient [39–41]. Besides, the improvement of the solution quality is not guaranteed by employing this approach.

Over the past years, performance metrics have been used to gauge the quality of solution sets produced by algorithms for MO problems [42, 43]. These metrics reflect the algorithm's capabilities/performance in solving a MO problem. Different performance metrics measure different aspect of the algorithm's capabilities, for instance, convergence, diversity, uniformity, and degree of dominance of solution spread (in a MO setting).

Hence, performance metrics have been used extensively in gauging solution quality. However, is it possible to further improve or modify an algorithm based on the assessment results produced by the performance metrics on a particular solution set? Secondly, if such a modification is possible, how does one go about using this information produced by the performance metrics? Assuming the answer to the first question is yes, then to answer the second question the information produced by the performance metrics need to be identified and classified. For this classification, the Hierarchy Axiom is postulated. Nevertheless, to increase the strength of this axiom an attempt is made to construct it from the “No Free Lunch (NFL) Theorems [44]”.

As observed in the NFL Theorems' [44], algorithm performances are very problem-dependent. These theorems indicate that over a large set of problems, the average performance of any pair of algorithms across all possible problems is identical. In simpler terms, if some algorithm's performance is better than another algorithm over some set of optimization problems, then the reverse must be true over the set of all other optimization problems. Therefore, the performance of a given algorithm is closely dependent on the “structure of the problem” tackled by it.

To keep things clear, let this “structure of the problem” be defined as “problem morphology.” Assuming that in MO scenarios, the problem morphology can be attained by analyzing the solution sets produced by a given algorithm. The results of this analysis can be obtained by the performance metrics. Thus, it can be stated that the results produced by performance metrics could be used to characterise and understand the problem morphology.

It is crucial to note that the definition of problem morphology introduced here is not the same as problem characteristics. Problem characteristic in an optimization context usually means the degree nonlinearity, the degree of nonconvexity, number of constraints, number of variables, and so forth. for a given problem. This sort of problem characteristics is commonly determined by observations done on the optimization problem formulation. “Problem morphology” on the other hand is used in this work to define the information which is numerically attained (results from the performance metrics) for the solution set of a given MO optimization problem. Hence, this distinguishes the “problem characteristics” from “problem morphology.” Although we have now established some logical connection between the problem morphology and the results produced by the performance metric, one question remains. How do we classify and use the information of the problem morphology to improve our algorithms? To attempt to answer this question, we now develop a formal postulate of the Hierarchical Axiom. In this argument, let us limit the number of metrics to three; say the convergence, diversity metrics, and the HVI. It is understood that the HVI gives us the degree of dominance of the solution sets, which in other words is our ultimate goal. The convergence and diversity metrics produces results that may be considered secondary to our ultimate goal. Since the results produced by all these metrics are from the same solution set, hence they must be connected in some unknown mathematical sense. Hence, our ultimate goal (solution set dominance) is influenced by the diversity and convergence metrics in an unknown manner. This unknown relationship would vary from problem to problem. This is due to the fact that the problem morphology also varies depending on the problem. This work proposes that by analysing the problem morphology by using the Hierarchy Axiom as a stepping point, this unknown relationship may be known.

**Figure 1:**
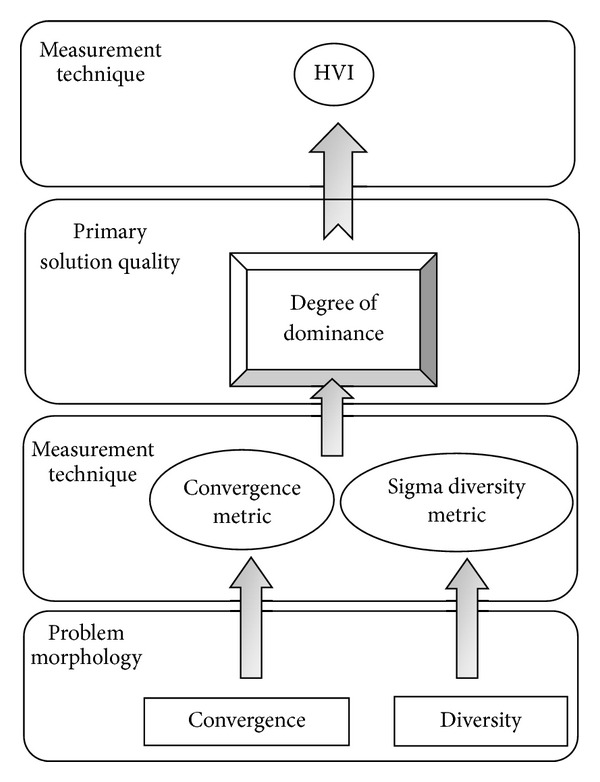
Hierarchy Axiom.

The hierarchy of priorities such that the degree of diversity and convergence of the solution sets directly influence the degree of dominance (measured by the HVI) is postulated. A hierarchical diagram representing this assumption is shown in Figure 1.

The summary of the ideas proposed thus far is that, performance metrics does not only measure the algorithm's performance, but due to the NFL theorems, they also measure the structure of the objective space. Thus, it can be said that performance metrics also describes the problem morphology of any given MO problem.

Since algorithms and problems are connected as described by the NFL theorems, the method to measure the problem morphology (namely, similar to measuring the algorithm's performance) is done by analyzing the solution set produced by any particular algorithm when tackling an MO optimization problem. Therefore, by testing a solution sets produced by a series of algorithms on a given MO problem using performance metrics, the problem morphology may be ascertained. In this work, the problem morphology includes the degree of convergence and diversity of the solution sets that depict the approximate Pareto frontier. As in most MO problems, the primary or critical solution quality here is defined by the degree of dominance of each solution set [15, 45]. Therefore, the primary solution quality is dependent on the problem morphology. Thus, it can be stated that the degree of dominance measured by the HVI is heavily influenced by the diversity and the convergence property of the solution set measured by the respective metrics. Based on these arguments, the following hypothesis is put forward. Since an algorithm's performance is problem-dependent, this performance is closely dependent on the morphology of the problem tackled by it. Performance metrics does not only measure the performance of any given algorithm; they inadvertently also measure the problem morphology. Therefore, by understanding the problem morphology, suitable adjustments or augmentations can be made to the algorithm to enhance the algorithm's capabilities.

**Figure 2:**
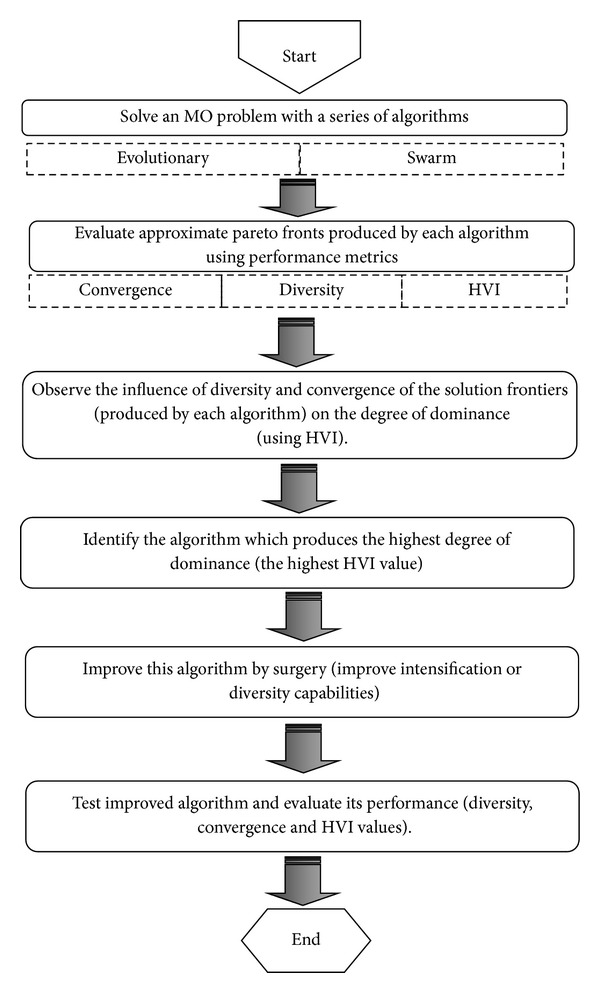
Proposed framework.

In the spirit of this philosophy, a framework of a solution method is proposed in this work to obtain the approximate Pareto frontier for MO problems.  Figure 2 shows the overall framework of the solution method.

First, a given problem is solved by using a series of metaheuristic methods. The degree of diversity, convergence, and HVI values is measured for all the approximate Pareto frontiers using performance metrics. Under the “Hierarchical Axiom,” the degree of convergence and diversity influences the degree of dominance. Thus, the influence of the convergence and the diversity metric values with respect to the HVI values of the approximate Pareto frontiers is analyzed. Next, the algorithm with the highest HVI value is identified. This algorithm is then improved based on the analysis done on the degree of convergence and diversity and their influence on the degree of dominance.

At this juncture, the concept of “surgery on algorithms” is further explained by employing an example. By obtaining the results that describe the problem morphology, the characteristic of the solution set (by implication, the characteristic of the algorithm) can be known. For instance, consider a scenario where a particular problem was tested with two algorithms, say Algorithms A and B. Algorithm A has produced a higher HVI value as compared to Algorithm B. In the sense of problem morphology, the solution set produced by Algorithm A is less diverse but more convergent compared to Algorithm B (see Table 2).

Hence, from the problem morphology standpoint, it can be stated that the degree of convergence influences the degree of dominance of the solution sets. This is due to the fact that Algorithm A is more dominant than B and has a higher degree of convergence. It is known from the Hierarchy Axiom that the problem morphology influences the degree of dominance. Therefore, it is possible to increase the degree of convergence of the solution sets hence directly affecting the degree dominance of those solutions by augmenting or modifying the algorithms. This kind of augmentation is defined in this work as “surgery on algorithms.” By performing surgery on Algorithm A, a new algorithm (say Algorithm C) would be generated that produces more convergent and more dominant solutions (see Table 3).

Since it has already been established (from Table 1), the convergence property is the one that influences the degree of dominance; the degree of diversity of Algorithms A and C is irrelevant and thus neglected.

It should be noted that the surgery on algorithms procedure is proposed in conjunction with the complete framework of the analysis of problem morphology and its consequent arguments and axioms. As opposed to hybridization, surgery on algorithms merely removes, adds, or modifies components in the algorithm based on the problem morphology. Therefore, the complexity of the algorithm does not increase radically and hence maintains its computational efficiency to a certain degree. However, hybridization often merges two whole algorithms into one. By doing so the complexity of the algorithms undergoes radical increment and this affects the computational efficiency of hybrid algorithms. Besides, by hybridizing algorithms, there is no guarantee that the hybrid algorithm produced may outweigh the results of its predecessors in their original form. These are the problems that may be addressed by employing the philosophical framework proposed in this work.

## 4. Mathematical Constructs

In this section, some definitions and axioms are presented in formalized mathematical forms. These forms are the foundation for the proposed framework put forth in this paper.

**Definition 1 (preliminaries)**

Let *A* ^*j*^ be a MO algorithm, and let *M* be a MO problem. The set *y*_*i*_ is defined as a set of decision variables to the problem *M*. The algorithm, *A* ^*j*^, operates on the set *y*_*i*_ and generates a set *x*_*i*_^*j*^ ∈ *P* ^*j*^ such that *x*_*i*_^*j*^ is a set of optimal points that construct the Pareto frontier *P* ^*j*^:

(2) $$\begin{array}{c} A^{j}:\left(y_{i}\in M\right)\to \left(x_{i}^{j}\in P^{j}\right), \end{array}$$

where *i* ∈ [1, *m*] denotes the number of optimal points and *m* is the maximal number of optimal points. The index *j* ∈ [1, *n*] denotes the individual solution set produced by algorithm *j*(*A*_*j*_) and *n* is the maximum number of algorithms applied.

**Definition 2 (metrics)**

The convergence and diversity metrics behave as an operator that maps the points in the solution sets, *x*_*i*_^*j*^ ∈ *P* ^*j*^ to a positive-definite real-valued scalar, *c* ^*j*^, *d* ^*j*^ ∈ *ℜ* ^+^. Let ∑ be the convergence metric, and let *δ* be the diversity metric such that

(3) $$\begin{array}{c} \sum :\left(x_{i}^{j}\in P^{j}\right)\to \left(c^{j}\in {ℜ}^{+}\right),\\ \delta :\left(x_{i}^{j}\in P^{j}\right)\to \left(d^{j}\in {ℜ}^{+}\right), \end{array}$$

for each *j* ∈ [1, *n*] and for all *i* ∈ [1, *m*].

**Definition 3 (hypervolume indicator)**

The hypervolume indicator (HVI) denoted Hyp maps the points in the solution sets, *x*_*i*_^*j*^ ∈ *P* ^*j*^, to a positive-definite real-valued scalar, dom⁡^*j*^ ∈ *ℜ* ^+^ where dom⁡^*j*^ is the degree of dominance. This mapping is given as follows:

(4) Hyp:(xij∈Pj)→(dom⁡j∈ℜ+) for  each  j∈[1,n]  ∀i∈[1,m].

The assumptions used for the metrics are the convergence, diversity, and dominance axioms. These statements are provided as follows.

**Axiom (convergence)**

Let *P* ^*j*^ and *P* ^*j*+1^ be two solution sets. If and only if *c* ^*j*^ > *c* ^*j*+1^, then the solution set *P* ^*j*^ is more convergent than *P* ^*j*+1^. If and only if *c* ^*j*^ > *c* ^*j*+1^, then the solution set *P* ^*j*+1^ is more convergent than *P* ^*j*^.

**Axiom (diversity)**

Let *P* ^*j*^ and *P* ^*j*+1^ be two solution sets. If and only if *d* ^*j*^ > *d* ^*j*+1^, then the solution set *P* ^*j*^ is more diverse than *P* ^*j*+1^. If and only if *d* ^*j*^ > *d* ^*j*+1^, then the solution set *P* ^*j*+1^ is more diverse than *P* ^*j*^.

**Axiom (dominance)**

Let *P* ^*j*^ and *P* ^*j*+1^ be two solution sets. If and only if dom⁡^*j*^ > dom⁡^*j*+1^, then the solution set *P* ^*j*^ is more dominant than *P* ^*j*+1^. If and only if *d* ^*j*^ > *d* ^*j*+1^, then the solution set *P* ^*j*+1^ is more dominant than *P* ^*j*^.

**Axiom (hierarchy)**

For any problem *M*, let *P* ^*j*^ be some solution sets produced by a mapping by *A* ^*j*^. Then, the values *c* ^*j*^ and *d* ^*j*^ influence the values dom⁡^*j*^ and not vice versa.

The problem morphology is then defined as follows.

**Definition 4 (problem morphology)**

For any problem *M*, let *P* ^*j*^ be some solution sets produced by a mapping by *A* ^*j*^. Then, the known values *c* ^*j*^, *d* ^*j*^, and dom⁡^*j*^ ∈ *ℜ* ^+^ and their possible relationships are described as the “problem morphology.”

**Proposition 5 (morphological relations)**

**Figure 3:**
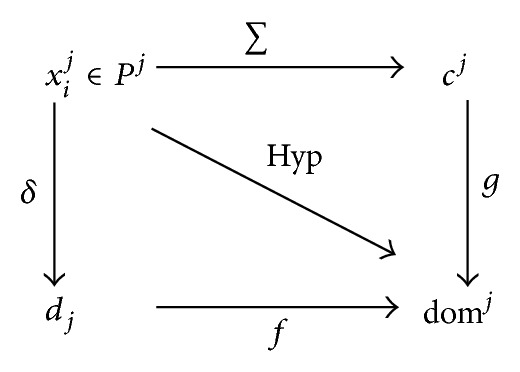
Graph representation of the morphological relations.

For any problem *M*, let *P* ^*j*^ be some solution sets produced by a mapping by *A* ^*j*^. Then the mappings by the metric operators, ∑,  *δ*, and Hyp can be described as in the graph diagram in Figure 3.

Thus, the unknown relations, *f* and *g*, describe the relationship between the degree of diversity and convergence with respect to the degree of dominance. The following relationships between the relations and the operators may be obtained as follows:

(5) $$\begin{array}{c} f\circ \delta =g\circ \sum =\text{Hyp}. \end{array}$$

**Axiom (numerical axiom)**

For any given problem *M*, the solution set *P* ^*j*^ is produced numerically by some algorithm, *A* ^*j*^. Thus, the performance of an algorithm, *A* ^*j*^, varies depending on the problem *M*. Therefore, *f* and *g* themselves which describe the problem morphology vary from problem to problem and cannot be obtained analytically. By implication, the problem morphology itself can only be obtained numerically by executing some algorithm *A* ^*j*^ on a problem, *M*, and getting some solution set *P* ^*j*^.

**Axiom (diversity and convergence mechanism)**

For any MO optimization problem *M*, the solution set, *P* ^*j*^, is produced numerically by some algorithm, *A* ^*j*^. The solution set, *P* ^*j*^, produced by *A* ^*j*^ has diversity and convergence values that can be computed using the ∑ and *δ* operators. There exist components *η* and *ξ* such that when incorporated into some algorithm, *A* ^*j*^, these components increase the diversity and convergence values of its solutions.

**Proposition 6 (surgery)**

Let an algorithm, *A* ^*j*^, be applied to some problem, *M*. The ∑, *δ*, and Hyp operators are applied to obtain the problem morphology. From the problem morphology, the relations *f* and *g* are established and two scenarios arise.

(1)
* The higher the value of c* ^*j*^ *, the higher the degree of dominance, dom* ^*j*^.
 

*For some problem, M, let the problem morphology be identified such that c* ^*j*^ ∝ *dom* ^*j*^ *. Then let A* ^*j*^ : (*y*_*i*_ ∈ *M*)→(*x*_*i*_^*j*^ ∈ *P* ^*j*^)*. Applying the operators *∑, *δ, and Hyp, the values c* ^*j*^, *d* ^*j*^, *and* *dom* ^*j*^ ∈ *ℜ* ^+^ *are obtained. If and only if an algorithm, A* ^*j*^ ⊗ *ξ* : (*y*_*i*_ ∈ *M*)→(*x*_*i*_^*j*+1^ ∈ *P* ^*j*+1^)*, is applied and if and only if for each j* ∈ [1, *n*]* and for all i* ∈ [1, *m*], ∑:(*x*_*i*_^*j*+1^ ∈ *P* ^*j*+1^)→(*c* ^*j*+1^ ∈ *ℜ* ^+^)* and *Hyp : (*x*_*i*_^*j*+1^ ∈ *P* ^*j*+1^)→(*dom* ^*j*+1^ ∈ *ℜ* ^+^)* is obtained, then c* ^*j*+1^ > *c* ^*j*^ *and* *dom* ^*j*+1^ > *dom* ^*j*^ *. The process of incorporating ξ into A* ^*j*^ *to develop the new algorithm A* ^*j*^ ⊗ *ξ is defined as surgery on the algorithm A* ^*j*^.
(2)
* The higher the value of d* ^*j*^ *, the higher the degree of dominance, dom* ^*j*^.
 

*Let M be some problem, and let the problem morphology be identified such that d* ^*j*^ ∝ *dom* ^*j*^ *. Let A* ^*j*^ : (*y*_*i*_ ∈ *M*)→(*x*_*i*_^*j*^ ∈ *P* ^*j*^)*. Applying the operators *∑, *δ, and Hyp, the values c* ^*j*^, *d* ^*j*^ *, and* *dom* ^*j*^ ∈ *ℜ* ^+^ *are obtained. If and only if an algorithm, A* ^*j*^ ⊗ *η* : (*y*_*i*_ ∈ *M*)→(*x*_*i*_^*j*+1^ ∈ *P* ^*j*+1^)*, is applied and if and only if for each j* ∈ [1, *n*]* and for all i* ∈ [1, *m*], *δ* : (*x*_*i*_^*j*+1^ ∈ *P* ^*j*+1^)→(*d* ^*j*+1^ ∈ *ℜ* ^+^)* and *Hyp : (*x*_*i*_^*j*+1^ ∈ *P* ^*j*+1^)→(*dom* ^*j*+1^ ∈ *ℜ* ^+^)* is obtained, then d* ^*j*+1^ > *d* ^*j*^ *and* *dom* ^*j*+1^ > *dom* ^*j*^ *. The process of incorporating η into A* ^*j*^ *to develop the new algorithm A* ^*j*^ ⊗ *η is defined as surgery on the algorithm A* ^*j*^.

Therefore, if any of these scenarios arises, then the components *η* and *ξ* may be incorporated into the algorithm, *A* ^*j*^, to obtain solutions with higher degree of dominance, *dom* ^*j*^, as mentioned above.

## 5. Numerical Experiments

The ideas proposed in this work were implemented to four industrial applications. The four applications were

- MO optimization of extraction process of bioactive compounds from *Gardenia* (Deep and Katiyar, 2010) [46];
- MO optimization of bioethanol production during cold enzyme starch hydrolysis (Bao et al., 2011) [47];
- MO optimization of synthesis gas production process (Mohanty, 2006) [48];
- MO optimization of pretreatment strategy for bioethanol production from rice husk (Banerjee et al., 2009) [49].

The MO problem presented in Deep and Katiyar, (2010) [46] (Application I) involves the optimization of the yields of certain chemical products which are extracted from the *Gardenia jasminoides* Ellis (J.E.) fruit. Chemical products such as crocins, geniposide, and the phenolic compounds (bioactive) are widely used in the food industry as natural food colorants (dyes). The phenolic compounds in *Gardenia* J.E. also have high antioxidant capabilities which make this fruit valuable for medicinal uses. This MO optimization model was for the extraction process of bioactive compounds from the *Gardenia* J.E. with respect to the constraints. The MO optimization model was developed to maximize the yield of three bioactive compound: crocin (in mg/g dry powder), geniposide (in mg/g dry powder), and total phenolic compounds (in mg/g dry powder) with respect to process parameters which are the concentration of ethanol in %, the extraction temperature in °C and the extraction time in minutes.

In Application II, the MO optimization of bioethanol production from cold enzyme starch hydrolysis is considered. The MO optimization of the fermentation process parameters is crucial for the successful production of ethanol from starch in an efficient and economical way. In Bao et al., 2011 [47], response surface methodology (RSM) was employed to model and optimize the cold enzyme starch hydrolysis conditions. In this application, the normalized objective functions are the predicted biomass in ×108 cells/mL, ethanol concentration in weight %, and starch utilization ratio in %. These objectives are to be maximized by selecting the optimal reaction parameters which are the amount of alpha-amylase (IU per g starch), the amount of glucoamylase (IU per g starch), the liquefaction temperature (°C), and the liquefaction time (in minutes).

As for Application III, the MO optimization of the noncatalytic combined reforming process was performed. A MO model was developed in Mohanty, 2006 [48], for the production of syngas/synthesis gas (CO + H_2_) by combined reforming. In combined reforming, two techniques which are Partial Oxidation of Methane Method (noncatalytic) and Steam Reforming (catalytic) are combined. The MO optimization model was developed based on responses from an experimental reactor setup. The normalized objective functions are the methane conversion in (%), carbon monoxide selectivity in (%), and hydrogen to carbon monoxide ratio as presented in Mohanty, 2006 [48]. These objectives were optimized with respect to the process parameters which are the oxygen to methane ratio (gmol/gmol), the hourly space velocity (h^−1^), and the reaction temperature (°C).

Finally the optimization of the pretreatment strategy for ethanol production from rice husk was used as Application IV. The pretreatment technique optimized in the work of Banerjee et al., 2009 [49], is the “Wet Air Oxidation” technique. This technique consumes very low amounts of fuel and its low in terms of operation costs. This method is a potentially effective pretreatment technique for fractionating lignocellulose into a solubilised hemicellulose fraction and a solid cellulose rich fraction with minimum inhibitor formation. In Banerjee et al., 2009 [49], the data from experiments were used to build a MO optimization model through multiple regression analysis. The objective functions which are cellulose yield in (%), lignin removal in (%), and hemicelluloses solubilisation in (%) were modelled in Banerjee et al., 2009 [49], with respect to the process parameters which were the reaction temperature (°C), the air pressure (MPa), and the reaction time (in minutes).

For Applications I and II, the proposed framework was carried out and surgery was performed on the PSO and DE algorithms, respectively. This was done by embedding the Hopfield component that enhances the convergence capabilities of the PSO and DE algorithms. The new algorithms generated are the HoPSO and HoDE. These results of the new algorithms are then compared with the original algorithm. The results comprising of the HVI, convergence, and diversity values of the entire Pareto frontier obtained by the methods are shown in Tables 4 and 5.

**Figure 4:**
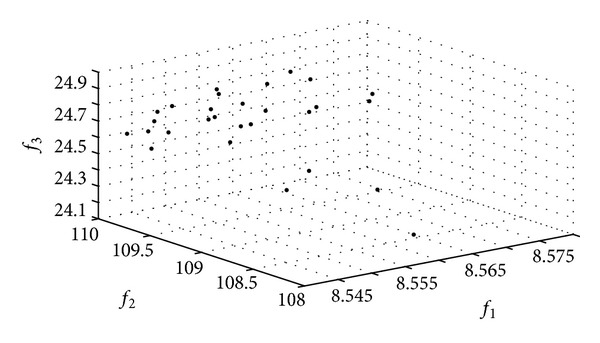
The Pareto frontiers of the HPSO algorithm.

**Figure 5:**
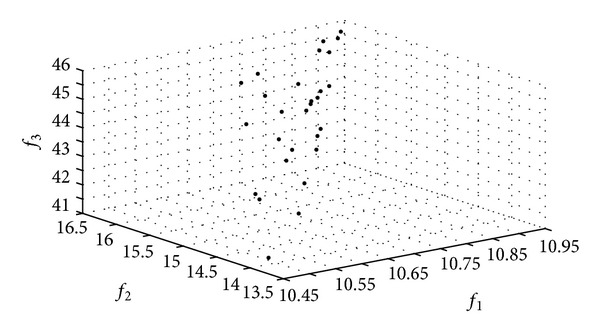
The Pareto frontiers of the HDE algorithm.

The Pareto frontier constructed by the HoPSO and the HoDE algorithms in Applications I and II are shown in Figures 4 and 5.

As for Application III and IV, the proposed framework was also implemented and surgery was carried out on the PSO and DE algorithms, respectively. This was done by embedding the chaotic component that enhances the diversity capabilities of the PSO and DE algorithms. The new algorithms generated are the CPSO and CDE. The computational results comprising of the HVI, convergence and diversity values of the entire Pareto frontier obtained by these methods are shown in Tables 6 and 7.

**Figure 6:**
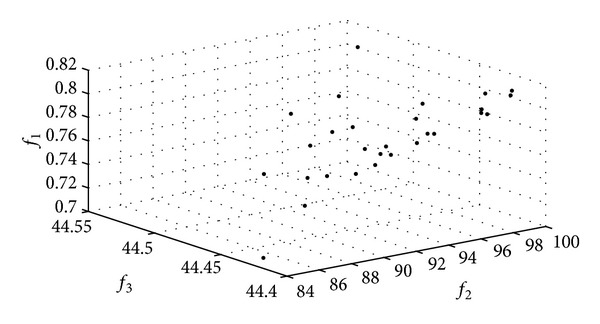
The Pareto frontiers of the CPSO algorithm.

**Figure 7:**
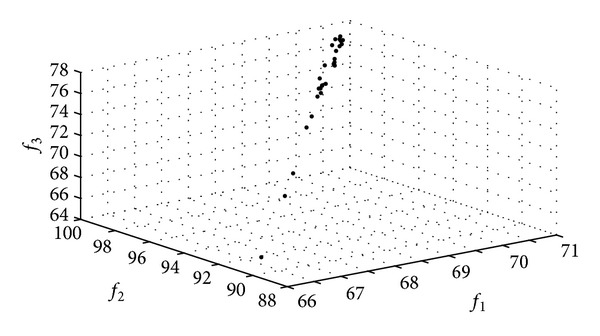
The Pareto frontiers of the CDE algorithm.

The Pareto frontier constructed by the CPSO and the CDE algorithms in Applications III and IV are shown in Figures 6 and 7.

In Figures 4–7, it can be observed that the new algorithms developed using the concept of surgery are more dominant as compared with their original counterparts (higher HVI value). Hence, these numerical experiments prove that the concept of SoA framework is effective in developing algorithms that produce highly dominant solutions for Pareto frontier construction.

## 6. Conclusions and New Perspectives

In this work, we have provided an overview of the prevalent methods applied in MO optimization. Besides, we have also discussed some current research issues faced in this area. Based on the ideas proposed, one of the ways forward in MO optimization is to develop a general framework/procedure to solve three-objective (or more) problems. Besides, proceeding in this line of thought, new directions are revealed for studies involving the effects of convergence/diversity of the solution set on the degree of Pareto dominance produced by any given MO algorithm. These insights pave a new path to our understanding of structures and constructions of objective spaces.

By these studies, an alternative or an enhancement to the hybridization approach (the proposed idea of “surgery”) which produces algorithms which are computationally less intensive, robust, effective, and capable of generating high-quality solutions could be developed. Extensive analysis and comparative works of solutions produced by these algorithms with standard algorithms could be carried out. By these comparisons, the strengths and drawbacks of the proposed framework may be studied in more detail. Most importantly, despite solving MO problems efficiently, the views presented in this work also points to an angle of inquiry that would increase our understanding of the nature of MO problems.

## Figures and Tables

**Table 1:** Developments of MO optimization in industrial applications.

Reference	Application	Technique
Aguirre et al., 2004 [50]	A field programmable transistor array (FPTA)	Inverted shrinkable Pareto archived evolution strategy (ISPAES)
Reddy and Kumar, 2007 [51]	“Truss design (Palli, et al., 1999 [52])” “I-beam design (Yang et al., 2002 [53])” “Welded beam design (Deb et al., 2000 [54])”	MO particle swarm optimization (MOPSO)
Kusiak et al., 2010 [55]	HVAC	MOPSO
Van Sickel et al., 2008 [56]	Control optimization for power plant	Multiobjective evolutionary programming (MOEP) and MOPSO
Heo et al., 2006 [57]	Control optimization for fuel power plant	PSO variants
Song and Kusiak, 2010 [58]	Temporal process optimization	Hybrid data mining (DM) and evolutionary strategy algorithm
Gunda and Acharjee, 2011 [59]	Economic/environmental dispatch problem	Pareto frontier DE (PFDE)
King et al., 2005 [60]	Power generation	NSGA-II
Kehinde et al., 2010 [61]	Economic/environmental dispatch	Hybrid convergence accelerator and the NSGA-II (Adra et al., 2009 [62])
El-Wahed et al., 2008 [63]	Economic/environmental dispatch	Hybrid ant colony optimization (ACO) and the modified SIMPLEX method
Abido, 2003 [64]	Economic/environmental dispatch	Hybrid NSGA hierarchy clustering algorithm and the fuzzy theory
Ganesan et al., 2011 [65]	Mould systems materials engineering	Hybrid NBI and GA
Sankararao and Gupta., 2007 [66]	Industrial fluidized-bed catalytic cracking unit	Jumping gene MOSA or MOSA-Jg
Rajesh et al., 2000 [67]	Steam reformer performance optimization	Hybrid NSGA
Behroozsarand et al., 2009 [68]	Optimization of an industrial autothermal reformer	NSGA-II
Martinsa and Costa., 2010 [36]	Optimization of a benzene production process	MO simulated annealing (MOSA)
Salari et al., 2008 [37]	Optimization of an ethane thermal cracking reactor	NSGA-II
Fiandaca and Fraga, 2009 [38]	Design optimization of pressure-swing adsorption	Multiobjective GA (MOGA)

**Table 2:** Comparison of solution sets produced by Algorithms A and B.

Algorithm	Diversity	Convergence	HVI
A	Low	High	High
B	High	Low	Low

**Table 3:** Comparison of solution sets produced by Algorithms A and C.

Algorithm	Diversity	Convergence	HVI
A	Low	High	High
C	Low	Higher	Higher

**Table 4:** The HVI, convergence, and diversity values produced by HoPSO.

	HVI	Convergence metric value	Diversity metric value
PSO	643878	0.3779	0.35714
HoPSO	647080	0.06766	0.10714

**Table 5:** The HVI, convergence, and diversity values produced by HoDE.

	HVI	Convergence metric value	Diversity metric value
DE	176652	0.0776	0.03571
HoDE	206586	0.05074	0.10714

**Table 6:** The HVI, convergence, and diversity values produced by CPSO.

	HVI	Convergence metric value	Diversity metric value
PSO	131142	0.11455	0.14286
CPSO	143857	0.38192	0.28571

**Table 7:** The HVI, convergence, and diversity values produced by CDE.

	HVI	Convergence metric value	Diversity metric value
DE	1180123	0.04465	0.17857
CDE	1922933	0.02243	0.28571
